# Incidence of valvular regurgitation and leaflet perforation by using automated titanium fasteners (CORKNOT®) in heart valve repair or replacement: less usual than reported

**DOI:** 10.1186/s13019-021-01512-z

**Published:** 2021-06-07

**Authors:** Faizus Sazzad, Ong Zhi Xian, Ashlynn Ler, Chang Guohao, Kang Giap Swee, Theo Kofidis

**Affiliations:** 1 Cardiac Surgery Experimental Laboratory, Department of Surgery, Yong Loo Lin School of Medicine, National University of Singapore, Singapore, Singapore; 2 Department of Cardiac, Thoracic and Vascular Surgery, National University Heart Centre, 1E Kent Ridge Road, Singapore, 119228 Singapore; 3 Cardiovascular Research Institute, National University of Singapore, Singapore, Singapore; 4 School of Medicine, National University of Ireland, Galway, Ireland; 5 National University Hospital, National University Health System, Singapore, Singapore

**Keywords:** Automated fastener, Heart valves, Heart valve prosthesis, Sutures, Corknot

## Abstract

**Background:**

CORKNOT® facilitates a reduction in cardiopulmonary bypass (CPB) time, aortic cross clamp (ACC) time and operative time, but reported to be associated with other complications. We aim to quantify the incidence of valvular complications related to CORKNOT® and determine the feasibility of its use between different valvular surgeries.

**Methods:**

Patients who underwent heart valve repair or replacement surgery via the use of automated titanium suture fasteners (CORKNOT®) in a tertiary care hospital were included in the study. This single-centre retrospective study was conducted on 132 patients between January 2016 and June 2018.

**Results:**

In our study, the overall mean operative time was 320.0 ± 97.0 min, mean CPB time was 171.4 ± 76.0 min and the calculated mean ACC time was 105.9 ± 54.0 min. Fifty-eight patients (43.9%) underwent minimally invasive valve replacement or repair surgery and 66 patients (50.0%) underwent concomitant procedures. A total of 157 valves were operated on, with 112 (84.8%) single valve surgeries, 15 (11.4%) double valve surgeries and 5 (3.8%) triple valve surgeries. After reviewed by the cardiologist blinded towards the study, we report trivial and/or mild paravalvular leak (PVL) in immediate post-operative echocardiography was found in 1 (1.01%) patients. There were no reported cases of valvular thrombosis, leaflet perforation, device dislodgement or embolization, moderate and/or severe PVL during hospitalization and follow-up echocardiography within 1 year. Single mitral valve and aortic surgeries had comparable incidences of post surgical complications.

**Conclusion:**

We conclude the feasibility of CORKNOT® utilisation in mitral and aortic valve surgeries. Additionally, incidence of CORKNOT® related complications in heart valve repair or replacement surgery is less usual in our setting than previously reported. These results motivate the use of CORKNOT® as a valid alternative with complete commitment.

**Supplementary Information:**

The online version contains supplementary material available at 10.1186/s13019-021-01512-z.

## Introduction

The CORKNOT® device is an automated titanium suture fastening system used predominantly in minimally invasive cardiac surgery. The CORKNOT® device has been reputed for its benefits in terms of strength, security and reliability [1, 2]. It is an ergonomic device that allows automatic crimping and trimming of sutures with a single squeeze. It enables suture orientation away from the valvular leaflets while allowing simultaneous suture tensioning and prosthetic cuff compression for suture security.

CORKNOT® was shown to be consistently better compared to hand-tied knot, resulting in a reduction in cardiopulmonary bypass time, aortic cross clamp time and operative time reported in minimally invasive mitral valve repair and aortic valve replacement [3]. Comparisons between CORKNOT® and the standard hand-tied sutures have shown that suture strength, consistency and speed to be superior [4]. The relative strengths of CORKNOT® are evident, but the correlation with valvular complications requires further inquiry.

However, overall procedural time savings do come with the additional cost of the more expensive automated titanium suture fastener [5]. Valvular regurgitation associated with the use of CORKNOT® has also been reported [6]. Delayed metallic embolization of the CORKNOT® fasterner [7] may also indicate the need for long-term surveillance in patients.

The surge in the popularity of CORKNOT® as an efficacious tool in supporting the rise of minimally invasive cardiac procedures warrants assessment of its risks and benefits. This study was designed to investigate the incidence of valvular complications in patients where COR-KNOT® was used and draw preliminary conclusions on the risks involved in using automated titanium suture fasteners in cardiac valve replacement and/or repair surgeries.

## Methods

### Study participants

This is retrospective cohort study was approved by the Domain Specific Review Board (DSRB) under National Healthcare Group (No# 2018/01269), Singapore. One hundred thirty-six patients who underwent heart valve repair or replacement surgery via automated titanium suture fasteners (CORKNOT®) in the Department of Cardiothoracic Surgery in a tertiary care hospital between January 2016 through June 2018 were analysed. Patients who underwent single, double or triple valvular surgeries were included in the study. One hundred thirty-two patients met the inclusion criteria. Patients who had missing valvular surgery parameters and post-operative echocardiographic variables were excluded from the study. One patient had a sutureless prosthesis failure and required on-table revision with sutured bioprosthesis. Patients were stratified by the number of valves operated on (single vs double vs triple) and the type of valve operated on (aortic vs mitral vs tricuspid vs pulmonary). The overall study population was divided into replacement and repair group, which entails 99 patients enrolled into the replacement group whereas 33 patients were in repair group.

### Outcome measurements and ascertainment

Baseline characteristics, peri-operative parameters, and follow-up parameters of patients were extracted from the local electronic database from our institute. The primary outcomes were post-operative ejection fraction, paravalvular leak, transvalvular regurgitation and valvular thrombosis assessed via post-operative echocardiography. The secondary outcomes consisted of re-interventions, cardiac mortality and all-cause mortality.

### Statistical analysis

Continuous data were presented as mean with standard deviation. Categorical data were presented as frequencies and percentages. Hypothesis testing via Chi-square test and Student’s t-test were utilised for categorical and continuous variables respectively. Statistically significant variables are defined as *p* < 0.05. All statistical analyses were performed with IBM SPSS Statistics Version 23.0 [IBM Corporation (2015)].

## Results

The final study population consisted of 132 patients who underwent heart valve replacement and/or repair procedures using CORKNOT®. Patients who had co-morbidities, active endocarditis *(15, 11.4%)*, undergoing concomitant procedures *(66, 50.0%)* or had undergone cardiac surgery previously *(10, 7.6%)* were included in this study to improve the internal validity of the study. The overall summary of the results is compiled in supplementary Table-S1.

### Patient characteristics and peri-operative parameters of all patients

Baseline characteristics of patients in both repair and replacement group are summarised in Table 1. The patients were predominantly male (69.4%) and Chinese (72.4%) with a mean age of 60 ± 12.3 years. Patients who underwent single mitral valve surgery (SMV) had a lower mean age (SMV: 57.9 ± 11.7 vs SAV: 62.9 ± 12.5, *p* < 0.05), higher pre-operative ejection fraction (%) (SMV: 58.8 ± 11.3 vs SAV: 52 ± 13.5, *p* < 0.05) and lower proportion of patients with hyperlipidemia (SMV: 28 (42.4%) vs SAV: 30 (69.8%), *p* < 0.05) and chronic lung disease. (SMV: 1 (1.5%) vs SAV: 5 (11.6%), *p* < 0.05) compared to patient who underwent single aortic valve surgery (SAV). Other existing comorbidities were comparable between both groups, except for hyperlipidemia and chronic lung disease (Table 1).

**Table 1:** Baseline preoperative characteristics of patients who underwent valve surgery via the use of CORKNOT®

Demographics	Subclass distribution					Comparison group		
Variables	Valve Replacement (*n* = 89)	Combined Repair and replacement (*n* = 10)	Valve Repair (*n* = 33)	Single Mitral Valve Repair (*n* = 29)	Double Valve Repair (*n* = 4)	Single Mitral Valve Replacement (*n* = 37)	Single Aortic Valve Replacement (*n* = 43)	*p*-value
Age – year (mean ± SD)	61.2 ± 12.0	66.6 ± 10.9	55.1 ± 12.2	54.6 ± 12.6	58.8 ± 9.5	60.5 ± 10.3	62.9 ± 12.5	0.357
Gender						19/37	34/43	0.009
Male	59 (66.3%)	6 (60%)	26 (78.8%)	23 (79.3%)	3 (75%)	19 (51.4%)	34 (79.1%)	
Female	30 (33.7%)	4 (40%)	7 (21.2%)	6 (20.7%)	1 (25%)	18 (48.6%)	9 (20.9%)	
Ethnicity						26/37	32/43	0.031
Chinese	65 (73.0%)	8 (80%)	22 (66.7%)	19 (65.5%)	3 (75%)	26 (70.3%)	32 (74.4%)	
Malay	5 (5.6%)	0	3 (9.1%)	3 (10.3%)	0	4 (5.0%)	4 (9.3%)	
Indian	5 (5.6%)	0	4 (12.1%)	4 (13.8%)	0	5 (6.3%)	1 (2.3%)	
Caucasian	3 (3.4%)	0	–	–	0	3 (3.8%)	3 (7.0%)	
Others	11 (12.4%)	2 (20%)	4 (12.1%)	3 (10.3%)	1 (25%)	7 (18.9%)	3 (7.0%)	
Body Surface Area – m2 (mean ± SD)	–	–	1.81 ± 0.19	1.8 ± 0.2	–	1.73 ± 0.25	1.82 ± 0.21	0.129
Pre-operative Ejection Fraction – % (mean ± SD)	55.0 ± 12.0	59.0 ± 5.2	56.2 ± 14.4	57.8 ± 13.0	45.0 ± 21.2	59.5 ± 9.8	52.0 ± 13.5	0.007
Co-morbidities								
Stroke	5 (5.6%)	0	0	0	0	2 (5.4%)	2 (4.7%)	1.00
Transient Ischemic Attack	1 (1.1%)	0	0	0	0	0	1 (2.3%)	1.00
Congestive Cardiac Failure	16 (18.0%)	1 (10%)	3 (9.1%)	2 (6.9%)	1 (25%)	8 (21.6%)	5 (11.6%)	0.244
Hypertension	45 (50.6%)	5 (50%)	16 (48.5%)	14 (48.3%)	2 (50%)	14 (37.8%)	25 (58.1%)	0.078
Hyperlipidemia	47 (52.8%)	3 (30%)	14 (42.4%)	12 (41.4%)	2 (50%)	16 (43.2%)	30 (69.8%)	0.023
Peripheral Arterial Disease	7 (7.9%)	0	0	0	0	3 (8.1%)	3 (7.0%)	1.00
Myocardial Infarction	9 (10.1%)	1 (10%)	2 (6.1%)	2 (6.9%)	0	3 (8.1%)	6 (14.0%)	0.494
Diabetes	23 (25.8%)	1 (10%)	6 (18.2%)	4 (13.8%)	2 (50%)	10 (27.0%)	12 (27.9%)	1.00
Deep Vein Thrombosis	0	0	0	0	0	–	–	–
Pulmonary Embolism	0	0	0	0	0	–	–	–
Smoking	17 (19.1%)	0	5 (15.2%)	5 (17.2%)	0	6 (16.2	8 (18.6%)	1.00
Chronic Renal Disease	5 (5.6%)	0	1 (3.0%)	1 (3.4%)	0	5 (6.3%)	2 (4.7%)	0.658
Creatinine Clearance – ml/min (mean ± SD)	81.8 ± 26.6	75.5 ± 24.5	87.6 ± 19.5	88.7 ± 20.2	80.0 ± 12.6	82.1 ± 26.1	81.8 ± 26.1	0.960
Chronic Liver Disease	0	1 (10%)	0	0	0	–	–	–
Chronic Lung Disease	6 (6.7%)	0	1 (3.0%)	0	0	1 (2.7%)	5 (11.6%)	0.209
Active Endocarditis	14 (15.7%)	1 (10%)	0	0	0	10 (27.0%)	3 (7.0%)	0.030
Previous Cardiac Surgery	7 (7.9%)	3 (30%)	0	0	0	3 (8.1%)	3 (7.0%)	1.00
Poor Mobility	10 (11.2%)	4 (40%)	1 (3.0%)	1 (3.4%)	0	6 (16.2%)	4 (9.3%)	0.501

**Fig. 1:**
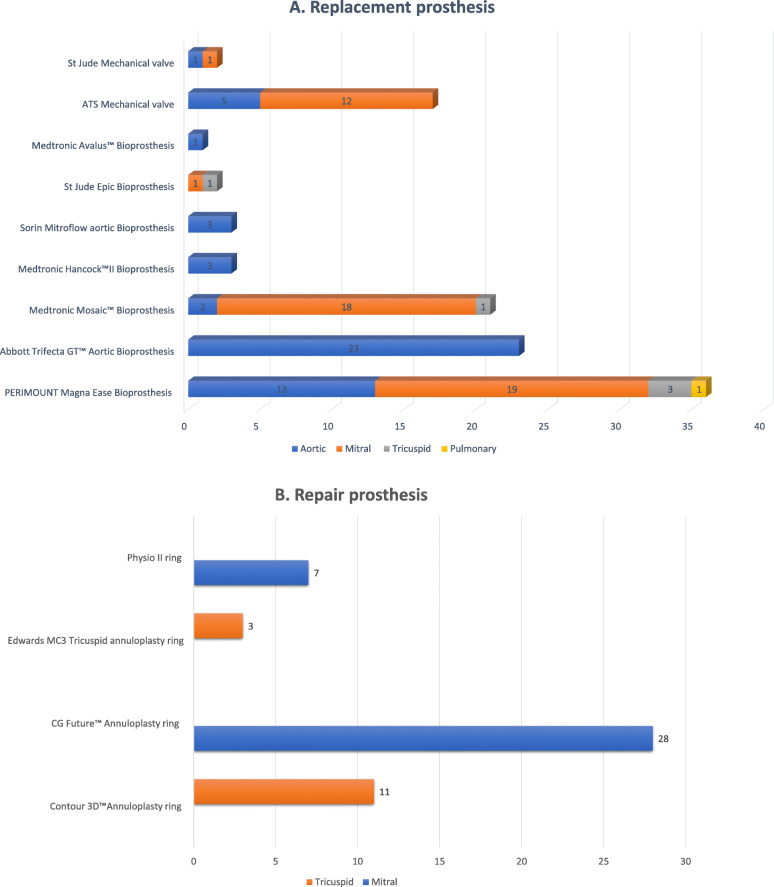
Distribution of all heart valve **a**. replacement prosthesis and **b**. annuloplasty repair rings used in the study population

Peri-operative parameters including follow up variables of patients in both repair and replacement group are summarised in Table 2. Overall, 58 patients (43.9%) underwent minimally invasive valve surgery and 74 patients (56.1%) had open heart valve surgery. Single valve surgeries were most common (112, 84.8%). Patients who underwent double valve surgery (15, 11.4%) and triple valve surgery (5, 3.8%) were few in comparison (supplementary Table-S1). The procedural distribution of the 157 operated prosthesis has been summarised in Fig. 1.

**Table 2:** Peri-operative and followup parameters of patients who underwent valve surgery via the use of CORKNOT®

Characteristics	Subclass distribution					Comparison group		
Variables	Valve Replacement (*n* = 89)	Combined Repair and replacement (*n* = 10)	Valve Repair (*n* = 33)	Single Mitral Valve Repair (*n* = 29)	Double Valve Repair (*n* = 4)	Single Mitral Valve Replacement (*n* = 37)	Single Aortic Valve Replacement (*n* = 43)	*p*-value
Minimally Invasive Surgery	31 (34.8%)	2 (20%)	25 (75.8%)	22 (75.9%)	3 (75%)	17 (45.9%)	13 (30.2%)	0.170
Double Valve	5 (5.6%)	6 (60%)	4 (12.1%)	–	–	–	–	–
Triple Valve	1 (1.1%)	4 (40%)	–	–	–	–	–	–
Operative Time – minutes (mean ± SD)	317.4 ± 94.0	369.9 ± 132.6	311.7 ± 91.8	311.2 ± 94.2	315.5 ± 83.3	304.4 ± 74.7	311.0 ± 94.9	0.736
CPB Time – minutes (mean ± SD)	168.3 ± 73.4	219.5 ± 95.7	164.8 ± 73.6	165 ± 78.1	163.3 ± 32.7	157.1 ± 68.0	167.2 ± 73.8	0.529
ACC Time – minutes (mean ± SD)	108.9 ± 55.5	120.4 ± 40.8	93.4 ± 52.6	94.4 ± 55.6	86.3 ± 27.2	96.3 ± 45.1	111.6 ± 58.0	0.204
Hospital stay – days (mean ± SD)	13.4 ± 11.7	21.8 ± 21.7	11.2 ± 5.4	11.3 ± 5.7	10.0 ± 2.2	14.1 ± 7.2	10.5 ± 11.6	0.018
ICU stay – days (mean ± SD)	4.3 ± 5.0	7.5 ± 6.1	3.8 ± 2.2	3.8 ± 2.0	3.0 ± 3.4	3.8 ± 1.9	3.4 ± 1.8	0.263
Chest Tube duration – days (mean ± SD)	4.9 ± 2.8	–	4.7 ± 1.4	4.6 ± 1.4	–	5.0 ± 2.3	4.1 ± 1.4	0.050
Immediate Post-operative Mild PVL or less^*a*^	3 (3.4%)	0	0	0	0	0	3 (7.0%)	0.245
Immediate Post-operative Significant PVL	2 (2.2%)	0	0	0	0	1 (2.7%)	1 (2.3%)	0.201
Stroke	0	0	0	0	0	0	0	–
Renal Failure	9 (11.1%)	2 (20%)	1 (3.0%)	1 (3.4%)	0	4 (10.8%)	4 (9.4%)	0.352
Atrial Fibrillation	29 (32.6%)	2 (20%)	13 (39.4%)	11 (37.9%)	2 (50%)	16 (43.2%)	9 (20.9%)	0.052
PPM Insertion^*b*^	6 (6.7%)	2 (20%)	4 (12.1%)	3 (10.3%)	1 (25%)	2 (5.4%)	3 (7.0%)	1.00
Bleeding/Transfusion	9 (10.1%)	2 (20%)	1 (3.0%)	0	1 (25%)	4 (10.8%)	2 (4.7%)	0.407
Thromboembolism	0	1 (10%)	0	0	0	0	0	0
Re-intervention	9 (10.1%)	0	2 (6.1%)	1 (3.4%)	1 (25%)	2 (5.4%)	3 (7.0%)	1.00
Cardiac Mortality	4 (4.5%)	0	0	0	0	1 (2.7%)	2 (4.7%)	1.00
All-cause Mortality	4 (4.5%)	0	0	0	0	1 (2.7%)	2 (4.7%)	0.505
Follow-up TTE								
Mild PVL or less		0	0	0	0	0	1 (2.3%)	-
Ejection fraction (%) (mean ± SD)	52.5 ± 11.4	53.- ± 10.1	52.8 ± 7.8	53.5 ± 7.8	47.5 ± 5.0	51.4 ± 10.0	53.7 ± 11.6	0.358
Thrombosis	0	0	0	0	0	0	0	–
Transvalvular regurgitation	4 (4.5%)	0	2 (6.1%)	1 (3.4%)	1 (25%)	2 (5.4%)	0	0.201

^a^*PVL* paravalvular leak, ^b^*PPM* Permanent pacemaker

Minimally invasive approach was more prevalent in patients who underwent single mitral valve surgery (SMV: 39 (59.1%) vs SAV: 13 (30.2%), *p* < 0.05). Patients who underwent single aortic valve surgery had shorter duration of chest tube insertion (SMV: 4.8 ± 1.9 vs SAV: 4.1 ± 1.4, *p* < 0.05). The proportion of concomitant procedures performed, operative time, cardiopulmonary bypass time, aortic cross clamp time, hospital stay and ICU stay were comparable between both groups (Table 2).

### Post-operative outcomes

The degree of paravalvular leak (trivial, mild, moderate or severe) was evaluated via post-operative transesophageal echocardiography (TEE) and/or transthoracic echocardiography (TTE). Two operated valves (2.02%) in replacement group had significant post-operative paravalvular leak. Reapplication of the aortic cross clamp and surgical revision in patients with significant post-operative paravalvular leak were routine. These two valves were re-evaluated to have post-operative trivial/mild paravalvular leak. Apart from the significant PVL, single valve showed mild postoperative PVL, which was not re-addressed surgically (Table 2).

All-cause mortality recorded were shown to be 4 (2.9%), out of which 1 (0.7%) was cardiac-related. Patients who underwent single mitral valve surgery had a higher incidence of new-onset atrial fibrillation (SMV: 27 (40.9%) vs SAV: 9 (20.9%), *p* < 0.05). Incidence of renal failure, permanent pacemaker insertion, bleeding events, reintervention and all-cause mortality were comparable between both groups (supplementary Table-S1).

### Follow-up outcomes

Majority of patients had follow-up between 3 and 12 months post-operatively (median follow*-*up time was ~ 4 months). There was no incidence of valvular thrombosis, device dislodgement and/or embolization or leaflet perforation recorded during follow-up. Trivial and/or mild paravalvular leak was incident in 1 operated valves (1.01%) while transvalvular regurgitation was incident in 4 operated valves (2.9%).

The mean ejection fraction (%) was lower compared to the pre-operative baseline measurement (52.6 ± 10.4 vs 55.6 ± 12.3). There was an improvement in ejection fraction on follow-up in patients who underwent single aortic valve surgery (52.0 ± 13.5 vs 53.7 ± 11.6). Overall, the incidence of paravalvular leak, transvalvular regurgitation and ejection fraction were comparable between patients who underwent single mitral valve surgery and single aortic valve surgery.

## Discussion

One of the basic skills of a surgeon is suturing and knot tying. To perform knot tying however, can be a tedious and time-consuming especially in minimally invasive heart valve surgery. This difficulty is largely related to the limited working space, limited degree of freedom for movement of surgical instruments. COR-KNOT® is an automated suture fastener recently proposed for valvular surgery. The COR-KNOT® device is designed to make suture fixation faster and to save operation time. A titanium occluder remotely and automatically secures sutures with a single squeeze of the built-in lever. At the same time the device also trims the excess suture tails [8].

Our study showed the feasibility of utilising CORKNOT® in both aortic and mitral valve surgeries. The incidence of paravalvular leak, transvalvular regurgitation and post-operative follow-up ejection fraction were comparable between both groups with comparable incidence with other studies. Similar conclusions regarding re-intervention, cardiac mortality and all-cause mortality both groups can be made. The difference in exposure and location of valves have proven to have minimal effect on valvular complications. The incidence of new-onset atrial fibrillation was observed to be higher in single mitral valve surgeries. However, atrial fibrillation is an expected complication of mitral valve surgeries due to the nature of valvular exposure compared to aortic valve surgery.

### Duration of operation

In this study, we observed a longer operative time compared to other studies. Grapow T.R. et al. [2] reported a mean operation time of 203.9 min (SD, 31.02), a shorter duration in comparison to our study. Various concomitant procedures were performed in 50% of our patients in the study; the complexity of concomitant operations may account for the difference in operative time. Our inclusion of patients with multiple comorbidities may prompt additional intra-operative considerations to ensure optimal post-operative recovery. Variability of surgical technique and level of experience of using CORKNOT® in our setting may contribute to this outcome.

### Transvalvular regurgitation

The safety and efficacy of COR-KNOT® was questioned in some cases of heart valve replacement surgery; especially with the use of bioprosthesis. Bracia and associates [6] postulated orientation of the COR-KNOT® Cor-Knot fastener resulted in transvalvular regurgitation in two of their reported cases. Balan et al. [9] reported a case of severe aortic regurgitation 8 months after implantation aortic valve prosthesis. On revision surgery the explanted valve had a perforation in alignment with one of the knots produced by the automatic knot fastener. Baciewicz et al. [10] also reported such case in their aortic valve series. In our practice we use COR-KNOT® to place correctly with a 180° twist of the wrist before deployment. This maneuver also been described by Biefer et al. [11], which ensures the orientation of the COR-KNOT®s away from the leaflets and prevents leaflet injury. Additionally, another potential mechanism of leaflet injury due to the use of a flexible (e.g. SJM Tailor flexible ring, St. Jude Medical) ring which is independent of the orientation of COR-KNOT®. The intrinsic flexibility of the ring may potentially rotate the ring inwards may lead to transvalvular mitral insufficiency [11]. In our series we didn’t use any flexible annuloplasty ring. Our series comprises Carpentier-Edwards Physio II annuloplasty ring and Edwards MC3 Tricuspid annuloplasty ring (Edwards Lifesciences), The Contour 3D™ annuloplasty ring (Medtronic). We postulate, the hypothesis of using rigid ring with COR-KNOT® may prevent leaflet injury is valid.

Transvalvular gradient and regurgitation is always a prime-concern especially during heart valve repair procedures [12]. In our series we had no such incident reported. Similarly, Morgant et al. [13] has observed no such complication as well in an 1:1 propensity matched prospective cohort. While looking at the mitral valve repair, which is now considered as standard of care for degenerative mitral valve pathology [14] use of COR-KNOT® is still not wide spread. Despite the fact, transvalvular regurgitation due to COR-KNOT® is sporadic and not observed in multiple trials. In a recent meta-analysis [15] looking at the use of the automated fastener vs hand-tied knots, a lower rate of postoperative valvular regurgitation (RR: 0.40, 95% CI:0.26 to 0.62, *p* < 0.0001) has been reported.

#### Paravalvular leak (PVL)

It is uncommon to have significant Paravalvular leaks (PVL) in most cases of heart valve replacement surgery but rarely can been observed as a complication of both mechanical and bioprosthetic valves.

Objective assessment of PVL may be challenging [15] and may require cardiovascular magnetic resonance (CMR) to quantify. However, Transesophageal echo is still the gold standard method for immediate post procedure assessment of heart valve repair or replacement [16]. Additionally, Patients with PVL may remain asymptomatic for long period and can be associated with heart failure, hemolysis and/or endocarditis [17]. PVL has been known to be associated with heart valve surgery, despite the use of traditional hand-tie [18, 19]

Moderate to severe paravalvular leak was reported to be present in the range of 3–6% in studies done by O’Rourke et. Al [20], Smolka, G [21]. and Kliger, C [﻿21﻿]. Hwang H.Y. et al. [﻿22﻿] reported an incidence of 4.5% of post-operative paravalvular leak. These studies were comparable to our incidence of post-operative paravalvular leak. However incidence of moderate to severe paravalvular leak in these studies was higher compared to our institution. The nil incidence of moderate to severe paravalvular leak is attributed to no incidences of patient-prosthetic mismatch and strict institutional protocol. Routine aortic cross clamp and revisions of significant paravalvular leak is an institutional practice, with the resolution of paravalvular leak in majority of cases during the early post-operative period. Trivial or mild paravalvular leak was present in minority of patients (5.1%) in our study. Consistent use of CORKNOT® may allow surgeons to progress through the learning curve at a quicker pace and achieve superior valvular replacement or repair outcomes.

#### Embolization

Metallic embolus that may be originated from the heart valve prosthesis, suture materials and/or CORKNOT® has been reported to the literature few times [7, 24]. Despite the fact that COR-KNOT® was proven in Ex-vivo model [4, 25] in terms of its sutures security, strength, and consistency; such detrimental rare event may happen.

The two cases reported the embolization by Garrett et al. [7] and Sagheer et al. [24] were not identical, the former was a robotic assisted procedure whereas the later was a traditional open heart surgery. Sagheer et al. [24] didn’t confirm the resulted embolus was CORKNOT® as the patient was believed to have paroxysmal positional vertigo, but the embolus had a metal density.

It may be possible that the resulted embolization from the robotic surgery case was due to inadequate knot security as a consequence of lake of tactile sensitivity. The incidence of embolization of CORKNOT® have not been reflected in clinical trials or larger scale studies. Similarly, our study reported nil incidence of leaflet perforation with bioprosthesis or repaired heart valves. Larger-scale comparative studies will be required to elicit the true complication profile of CORKNOT®.

#### In-hospital mortality and morbidity

Hwang H.Y. et al. [﻿22﻿, 23] and Pinheiro C.P. et al. [26] reported institutional in-hospital mortality rates of 6.1 and 4.5% respectively, both which showed poorer outcomes in comparison to our study. Postoperative complications reported by Beute T.J. et al. [3] included 1 incident of stroke (1.9%), 2 incidents (3.8%) of renal failure, 17 incidents (33.0%) of new-onset atrial fibrillation, 3 incidents (5.7%) of permanent pacemaker insertions and 11 incidents (21.2%) of post-operative transfusion of blood products. Except for renal failure and permanent pacemaker insertions, we observed the same findings in our own study, where COR-KNOT® patients had comparable rates of operative mortality, cardiovascular and neurological complications, and pulmonary and renal complications in all subgroup analyses.

### Follow-up outcome

A routine follow up may reveal the intermediate term results of the overall procedure, comprises a range of delayed complication. It is important to note, that though rare, cases of COR-KNOT®-related complications should not be overlooked. Echocardiographic evaluation of paravalvular leak and transvalvular regurgitation should be evaluated in relation to COR-KNOT® and record routinely.

Beute T.J. et al [3] reported a mean post-operative follow-up ejection fraction of 57.7% (SD, 12.2) in the automated titanium suture fasteners group, which was higher than the ejection fraction measured during follow-up in our study. In the same study, the mean pre-operative ejection fraction of the same group was 62%. The decline in ejection fraction in the study was 4.3%, comparable to the 4.0% reported in our study. The decline in ejection fraction from baseline to follow-up is therefore an expected outcome. Paravalvular leaks were observed to resolve either via on-table surgical revisions (if severe) or spontaneously (if mild to moderate) in the early post-operative period. Regardless, a small percentage of patients were identified to have trivial or mild paravalvular leak which persisted or developed during follow-up transthoracic echocardiography. Most reported trivial or mild paravalvular leaks were resolved during the follow-up, suggesting spontaneous resolution of paravalvular leak. However, 4 patients developed paravalvular leak between post-operative and follow-up. Additionally, our study reported no incidence of leaflet perforation with bioprosthetic heart valves and no re-interventions were required for paravalvular leak during follow-up. Larger-scale comparative studies will be required to elicit the true complication profile of CORKNOT®.

### Limitations

This is a single-centre retrospective database analysis which has relatively low case volume. Our analysis had a heterogenous patient population. There was a wide range of procedures, valve types, indications for surgery, and surgical approaches in our series, hence it was limited to single valve comparisons between commonly operated aortic and mitral valves. Analysis of multiple valves and less commonly operated valves such as pulmonary and tricuspid valves would be less feasible in our institution. Another limitation of our study is the paucity in current literature on complications arising from the use of automated titanium suture fasteners, with most being case reports or comments.

## Conclusions

With the surge and increasing preference towards minimally invasive cardiac procedures CORKNOT® may be an efficacious and facilitative tool. We have identified the feasibility in its utilisation between aortic and mitral valves. There is a potentially better outcome with the use of CORKNOT® given the low incidence of valvular complications observed and paravalvular leak compared to similar studies.

## Supplementary Information

**Additional file 1: Video S1.**

**Additional file 2: Figure S1.** Corknot preparation – mounting and loading of a single application occlude at the applicator tip during AVR via upper J mini sternotomy.

**Additional file 3: Figure S2.** Corknot application – Apply the metallic occlude while both the valve suture for a single stitch is held by the other hand. The sutures are to be in traction to avoid loose-tie.

**Additional file 4: Figure S3.** Corknot application – the operator’s hand needs to in 180^0^ rotation, once Corknot is delivered from this position the knot usually faces outwards and away from the leaflets.

**Additional file 5: Table S1.** Over all outcome in the study population.

## Data Availability

Deidentified research data is available on request.

## Abbreviations

CPB: Cardiopulmonary bypass
ACC: Aortic cross clamp
PVL: Paravalvular leak
SPSS: Statistical Package for the Social Sciences
SMV: Single mitral valve surgery
SAV: Single aortic valve surgery
ICU: Intensive care unit
TEE: Transesophageal echocardiography
TTE: Transthoracic echocardiography
SD: Standard deviation
