# Supplementary figures and images for: Incidence of valvular regurgitation and leaflet perforation by using automated titanium fasteners (CORKNOT®) in heart valve repair or replacement: less usual than reported
Source: J Cardiothorac Surg. 2021 Jun 7;16:163. doi: 10.1186/s13019-021-01512-z (PMC8186203; doi:10.1186/s13019-021-01512-z)

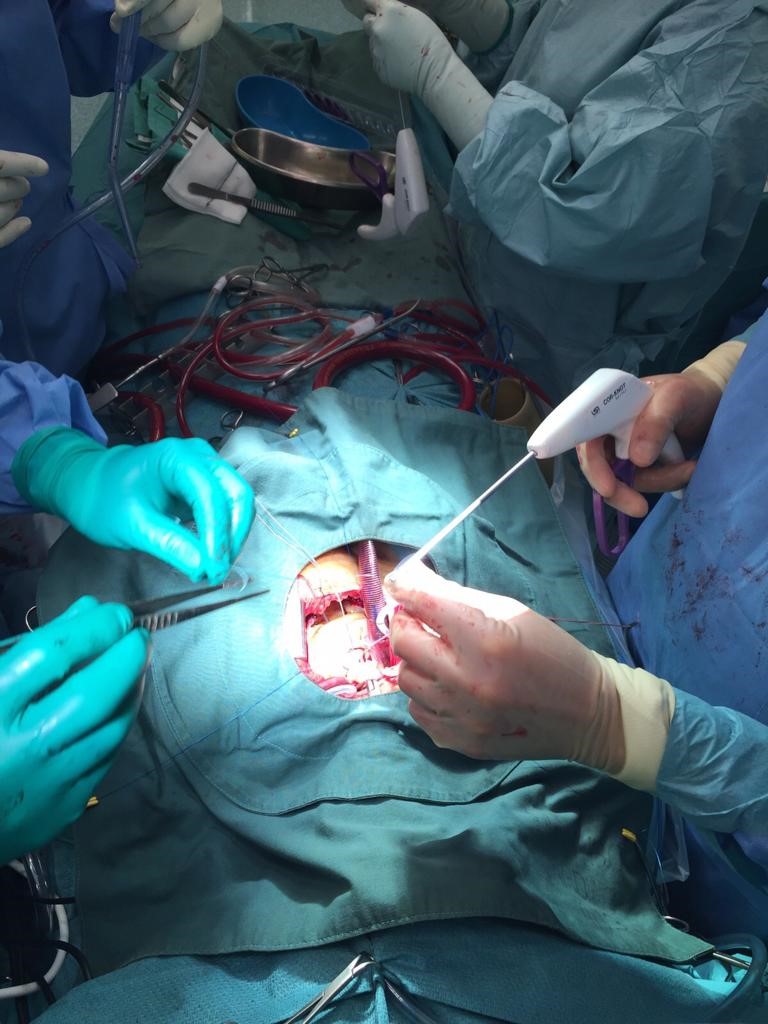

Supplement: Supplementary file 2 — Additional file 2: Figure S1. Corknot preparation – mounting and loading of a single application occlude at the applicator tip during AVR via upper J mini sternotomy. [file 13019_2021_1512_MOESM2_ESM.jpg]

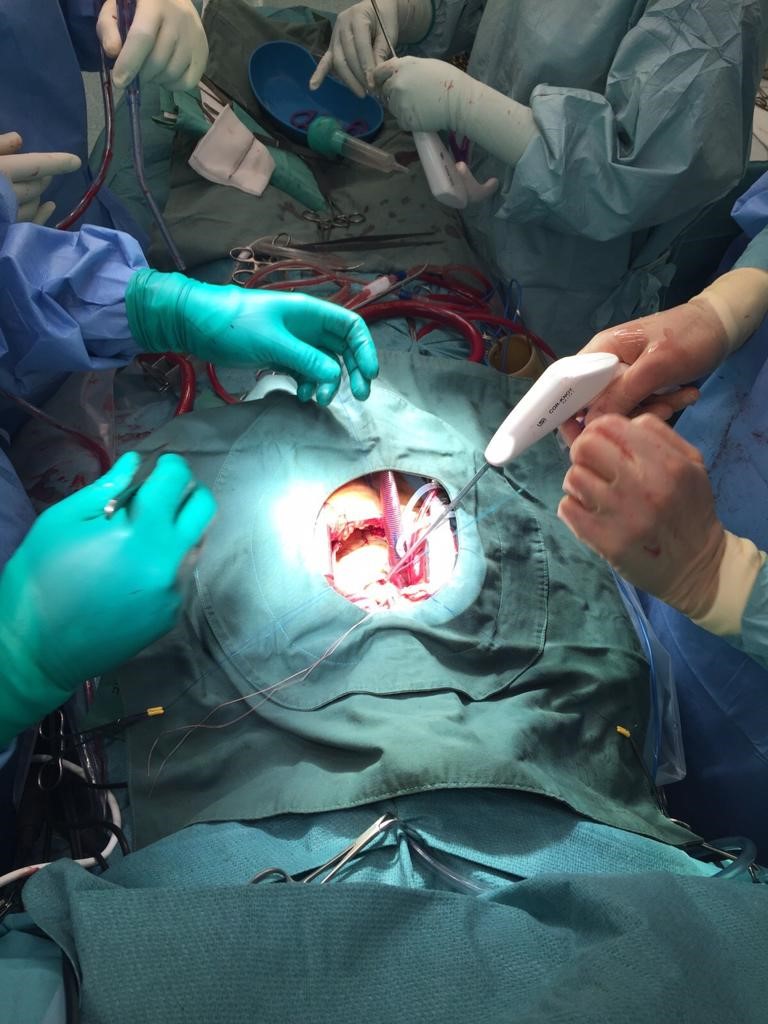

Supplement: Supplementary file 3 — Additional file 3: Figure S2. Corknot application – Apply the metallic occlude while both the valve suture for a single stitch is held by the other hand. The sutures are to be in traction to avoid loose-tie. [file 13019_2021_1512_MOESM3_ESM.jpg]

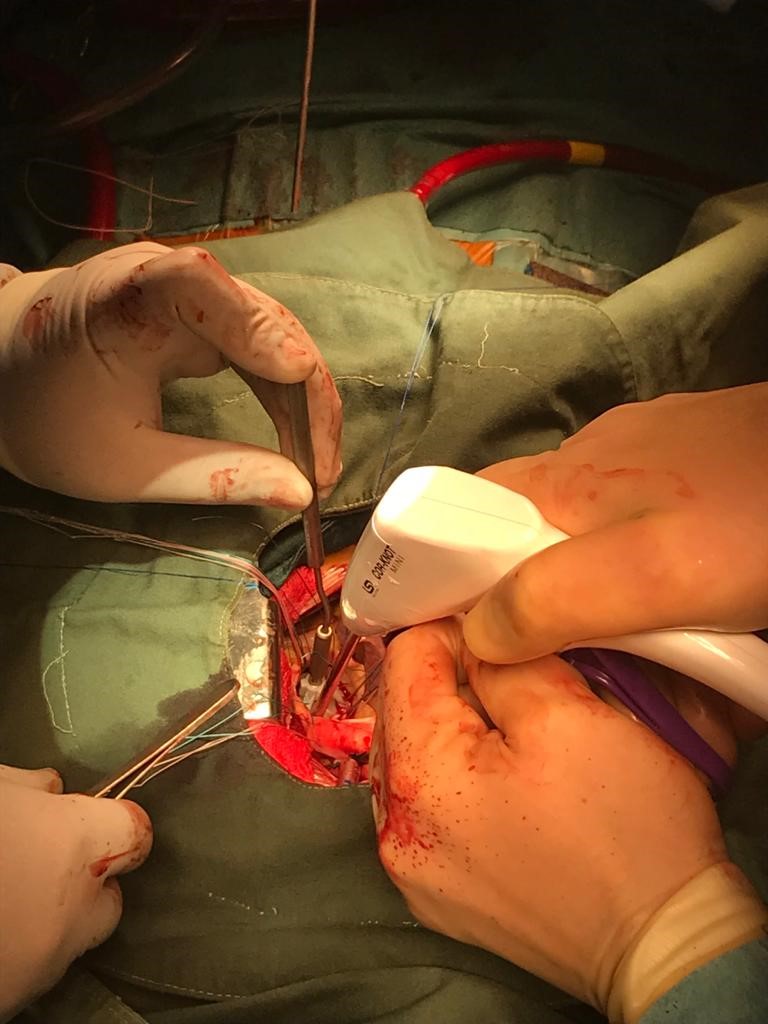

Supplement: Supplementary file 4 — Additional file 4: Figure S3. Corknot application – the operator’s hand needs to in 1800 rotation, once Corknot is delivered from this position the knot usually faces outwards and away from the leaflets. [file 13019_2021_1512_MOESM4_ESM.jpg]
