# Supplementary material for: Incidence of valvular regurgitation and leaflet perforation by using automated titanium fasteners (CORKNOT®) in heart valve repair or replacement: less usual than reported
Source: J Cardiothorac Surg. 2021 Jun 7;16:163. doi: 10.1186/s13019-021-01512-z (PMC8186203; doi:10.1186/s13019-021-01512-z)
Supplement: Supplementary file 5 — Additional file 5: Table S1. Over all outcome in the study population. [file 13019_2021_1512_MOESM5_ESM.docx]

**Supplementary Table 1** : Over all outcome in the study population

| **Baseline preoperative Characteristics** | **Patients  (n = 132)** | **Single Mitral Valve  (n = 66)** | **Single Aortic Valve (n = 43)** | **p-value** |
| --- | --- | --- | --- | --- |
| **Demographics** |  |  |  |  |
| Age – year (mean ± SD) | 60.1 ± 12.3 | 57.9 ± 11.7 | 62.9 ± 12.5 | < 0.05 |
| Gender  Male  Female | 91 (68.9%)  41 (31.1%) | 42 (63.6%)  24 (36.4%) | 34 (79.1%)  9 (20.9%) | 0.094 |
| Ethnicity  Chinese  Malay  Indian  Caucasian  Others | 95 (72.0%)  8 (6.1%)  9 (6.8%)  3 (2.3%)  17 (12.9%) | 45 (68.2%)  3 (4.5%)  8 (12.1%)  0  10 (15.2%) | 32 (74.4%)  4 (9.3%)  1 (2.3%)  3 (7.0%)  3 (7.0%) | <0.05 |
| Body Surface Area – m^2^ (mean ± SD) | 1.8 ± 0.2 | 1.76 ± 0.23 | 1.82 ± 0.21 | 0.233 |
| Pre-operative Ejection Fraction – % (mean ± SD) | 55.6 ± 12.3 | 58.8 ± 11.3 | 52.0 ± 13.5 | < 0.05 |
| **Co-morbidities** |  |  |  |  |
| Stroke | 5 (3.8%) | 2 (4.7%) | 2 (3.0%) | 0.646 |
| Transient Ischemic Attack | 1 (0.8%) | 0 | 1 (2.3%) | 0.394 |
| Congestive Cardiac Failure | 20 (15.2%) | 10 (15.2%) | 5 (11.6%) | 0.778 |
| Hypertension | 66 (50.0%) | 28 (42.4%) | 25 (58.1%) | 0.121 |
| Hyperlipidemia | 64 (48.5%) | 28 (42.4%) | 30 (69.8%) | <0.05 |
| Peripheral Arterial Disease | 7 (5.3%) | 3 (4.5%) | 3 (7.0%) | 0.679 |
| Myocardial Infarction | 12 (9.1%) | 5 (7.6%) | 6 (14.0%) | 0.337 |
| Diabetes | 30 (22.7%) | 14 (21.2%) | 12 (27.9%) | 0.493 |
| Deep Vein Thrombosis | 0 | 0 | 0 | - |
| Pulmonary Embolism | 0 | 0 | 0 | - |
| Smoking | 22 (16.7%) | 11 (16.7%) | 8 (18.6%) | 0.801 |
| Chronic Renal Disease | 6 (4.5%) | 3 (6.1%) | 2 (4.7%) | 1.00 |
| Creatinine Clearance – ml/min (mean ± SD) | 82.8 ± 24.9 | 85.0 ± 23.8 | 81.8 ± 26.1 | 0.521 |
| Chronic Liver Disease | 1 (0.8%) | 0 | 0 | - |
| Chronic Lung Disease | 7 (5.3%) | 1 (1.5%) | 5 (11.6%) | <0.05 |
| Active Endocarditis | 15 (11.4%) | 10 (15.2%) | 3 (7.0%) | 0.240 |
| Previous Cardiac Surgery | 10 (7.6%) | 3 (4.5%) | 3 (7.0%) | 0.679 |
| Poor Mobility | 15 (11.4%) | 7 (10.6%) | 4 (9.3%) | 1.00 |
| **Peri-operative parameters** |  |  |  |  |
| Minimally Invasive Surgery | 58 (43.9%) | 39 (59.1%) | 13 (30.2%) | <0.05 |
| Single Valve | 112 (84.8%) | - | - | - |
| Double Valve | 15 (11.4%) | - | - | - |
| Triple Valve | 5 (3.8%) | - | - | - |
| Concomitant procedures | 66 (50.0%) | 26 (39.4%) | 22 (51.2%) | 0.243 |
| Operative Time – minutes (mean ± SD) | 320.0 ± 97.0 | 307.4 ± 83.2 | 311.0 ± 94.9 | 0.836 |
| CPB Time – minutes (mean ± SD) | 171.4 ± 76.0 | 160.5 ± 72.1 | 167.2 ± 73.8 | 0.641 |
| ACC Time – minutes (mean ± SD) | 105.9 ± 54.0 | 95.5 ± 49.6 | 111.6 ± 58.0 | 0.130 |
| Hospital stay – days (mean ± SD) | 13.4 ± 11.8 | 12.9 ± 6.7 | 10.5 ± 6.0 | 0.065 |
| ICU stay – days (mean ± SD) | 4.1 ± 2.8 | 3.8 ± 1.9 | 3.4 ± 1.8 | 0.246 |
| Chest Tube duration – days (mean ± SD) | 5.0 ± 2.9 | 4.8 ± 1.9 | 4.1 ± 1.4 | 0.05 |
| **Post-operative complications** |  |  |  |  |
| Post-operative Moderate and/or Severe PVL | 2 (1.5%) | 0 | 2 (4.7%) | **-** |
| Post-operative mild PVL | 1 (0.75%) | 0 | 1 (2.3%) | **-** |
| Stroke | 0 | 0 | 0 | - |
| Renal Failure | 12 (9.1%) | 4 (6.1%) | 2 (4.7%) | 0.281 |
| Atrial Fibrillation | 44 (33.3%) | 27 (40.9%) | 9 (20.9%) | <0.05 |
| PPM Insertion | 6 (9.1%) | 5 (7.6%) | 3 (7.0%) | 1.00 |
| Bleeding/Transfusion | 12 (9.1%) | 4 (6.1%) | 2 (4.7%) | 1.00 |
| Thromboembolism | 1 (0.8%) | 0 | 0 | - |
| Re-intervention | 11 (8.3%) | 3 (4.5%) | 3 (7.0%) | 0.679 |
| Cardiac Mortality | 1 (0.7%) | 0 | 0 | - |
| All-cause Mortality | 4 (2.9%) | 1 (1.5%) | 2 (4.7%) | 0.561 |
| **Follow-up** |  |  |  |  |
| Moderate and/or Severe PVL | 0 | 0 | 0 | - |
| Follow-up mild PVL | 1 (0.75%) |  |  |  |
| Ejection fraction (%) | 52.6 ± 10.4 | 52.4 ± 9.1 | 53.7 ± 11.6 | 0.526 |
| Thrombosis | 0 | 0 | 0 | - |
| Transvalvular regurgitation | 6 (4.5%) | 3 (4.5%) | 0 | 0.174 |
